# Exosomes Immunity Strategy: A Novel Approach for Ameliorating Intervertebral Disc Degeneration

**DOI:** 10.3389/fcell.2021.822149

**Published:** 2022-02-10

**Authors:** Weihang Li, Shilei Zhang, Dong Wang, Huan Zhang, Quan Shi, Yuyuan Zhang, Mo Wang, Ziyi Ding, Songjie Xu, Bo Gao, Ming Yan

**Affiliations:** ^1^ Department of Orthopedic Surgery, Xijing Hospital, Air Force Medical University, Xi’an, China; ^2^ Department of Orthopaedics, Affiliated Hospital of Yanan University, Yanan, China; ^3^ Department of Critical Care Medicine, Xijing Hospital, Air Force Medical University, Xi’an, China; ^4^ The First Brigade of Basic Medical College, Air Force Military Medical University, Xi’an, China; ^5^ Beijing Luhe Hospital, Capital Medical University, Beijing, China

**Keywords:** exosome, intervertebral disc degeneration, immunologic therapy, vascularization, low back pain

## Abstract

Low back pain (LBP), which is one of the most severe medical and social problems globally, has affected nearly 80% of the population worldwide, and intervertebral disc degeneration (IDD) is a common musculoskeletal disorder that happens to be the primary trigger of LBP. The pathology of IDD is based on the impaired homeostasis of catabolism and anabolism in the extracellular matrix (ECM), uncontrolled activation of immunologic cascades, dysfunction, and loss of nucleus pulposus (NP) cells in addition to dynamic cellular and biochemical alterations in the microenvironment of intervertebral disc (IVD). Currently, the main therapeutic approach regarding IDD is surgical intervention, but it could not considerably cure IDD. Exosomes, extracellular vesicles with a diameter of 30–150 nm, are secreted by various kinds of cell types like stem cells, tumor cells, immune cells, and endothelial cells; the lipid bilayer of the exosomes protects them from ribonuclease degradation and helps improve their biological efficiency in recipient cells. Increasing lines of evidence have reported the promising applications of exosomes in immunological diseases, and regarded exosomes as a potential therapeutic source for IDD. This review focuses on clarifying novel therapies based on exosomes derived from different cell sources and the essential roles of exosomes in regulating IDD, especially the immunologic strategy.

## Introduction

Low back pain (LBP), one of the most severe medical and social problems globally, together with the causes of complete disability in middle-aged or older adults, has affected nearly 80% of the population worldwide. It is the most common cause of limited activity in patients younger than 45 (Taylor et al., 1994; Andersson, 1999; Millecamps et al., 2015). Dieleman et al. (2020) reported that the total cost of back pain, neck pain, and other musculoskeletal disorders comprised a great proportion of expenditures from 1996 to 2016—approximately $264 billion, which leads to considerable financial burdens on both society and families of affected individuals. IDD (intervertebral disc degeneration) is the main cause of LBP, and it occurs frequently in adults. It is a common musculoskeletal disorder; the progression results in disc herniation, spinal canal stenosis, and degenerative spondylolisthesis (Jin et al., 2013). While the exact etiology and degenerative mechanisms remain delineated, existing studies have discovered several factors involved in the initiation and progression of IDD, including aging, loading changes, poor nutrient supply, smoking, and hereditary aspects (Adams et al., 2000; Mannion et al., 2000; Freemont et al., 2001; Horner and Urban, 2001; Vogt et al., 2002; Bibby and Urban, 2004; Wang et al., 2012). Although etiology is likely multifactorial, mounting lines of evidence have pointed that genetic factor is considered as a pivotal risk of IDD, which accounts for more than 70%, with smaller contributions from environmental factors (Battié et al., 1995; Bijkerk et al., 1999; Sambrook et al., 1999; Kepler et al., 2013). The pathological basis of IDD includes the disorders of catabolism and anabolism in the extracellular matrix (ECM), continuous decrease in nucleus pulposus (NP) cells, and cellular and biochemical alterations in the microenvironment of intervertebral disc (IVD) (Wang et al., 2020a; Binch et al., 2021).

IVD, located between adjacent vertebrae, are fibrocartilaginous tissues and allow motion between vertebral bodies; they provide load support, flexibility, energy storage, and consumption in the spine (Ji et al., 2018). It is a complex avascular organ consisting of the central NP, peripheral annulus fibrosus (AF), which envelops the NP, and the upper and lower cartilage endplates (EP) (Le Maitre et al., 2007). A healthy NP is gelatinous and is primarily made of proteoglycans (glycosaminoglycan), type II collagen, and NP cells, while the peripheral AF is a thick, dense structure, and it is composed of type I collagen and AF cells; EPs seal the disc, which are cartilaginous structures that resemble the hyaline cartilage (Kadow et al., 2015). These distinct anatomical areas formed a special complex structure, giving it unique biomechanical properties to maintain spinal flexibility and mechanical stability (Lam et al., 2011). Mature IVD is made up of avascular tissues, which are inundated with extensive ECM; the blood supply through peripheral capillaries is rather limited, and nutrient supply could only be received from passive diffusion from the EPs (Boubriak et al., 2013). So, it is easily understood that IVD is prone to degeneration.

Currently, the therapeutic approaches regarding IDD mainly include conservative treatment and surgical intervention. Conservative treatment may not alleviate the patients’ pain immediately; patients could only maintain a normal life mainly through oral painkillers. Surgical interventions attempt to relieve symptoms rather than restore inherent structure and function. Discectomy is the most common spinal surgical treatment of IDD and typically performed in young patients about 25–40 years old, while the impact of the alteration in biomechanics and long-term sequelae may be significant (Hermantin et al., 1999; Yorimitsu et al., 2001). Brinckmann et al. found that loss of disc tissue resulted in a decrease in disc height and intradiscal pressure, and an increase in radial disc bulge (Brinckmann and Grootenboer, 1991). Scoville and Corkill (1973) reported a 50% incidence of narrowing after disc surgery at a 3-month follow-up. Tibrewal and Pearcy (1985) also found a significant narrowing of disc space following disc surgery, compared to non-operated controls. Patients suffer relapse after treatment, and the disk degeneration may even accelerate the degeneration of adjacent segments (Hashimoto et al., 2019), so recurrent disc herniation might be an inevitable issue for surgeons and patients; both oral pills and surgical methods could not considerably cure IDD. Consequently, further explorations about more effective IDD treatment approaches are of great significance. Studies have reported that the ideal strategy for disc regeneration is to restore the functions as well as the integrity of disc, including biomaterials therapy, native matrices supplement, mesenchymal stem cell (MSC) therapy, growth factors therapy, tissue engineering technology, immunotherapy, and exosome therapy (Longo et al., 2012; Jin et al., 2013; Richardson et al., 2016; Bowles and Setton, 2017; Cheng et al., 2018; Du et al., 2019; Sun et al., 2020a); these methods are currently the most compelling research avenues for IDD treatment.

Cell–cell communication is an essential way to exchange information between cells; paracrine signaling is the primary means of cellular communication, while exosome secretion is a special mechanism of paracrine regulation, which has been widely considered for IDD therapy (Lu et al., 2017). In this review, we focus on the novel approach of exosome therapy, to clarify the roles in regulating the immunological and inflammatory pathological process of IDD. This review provides a reference for elucidating the molecular mechanisms of exosomes in the treatment of IDD as well as its application prospects.

## Exosomes

Exosomes were firstly discovered in sheep reticulocytes by Pan and Johnstone in 1893. During the maturation of sheep reticulocytes, the release of transferrin receptors into ECM was correlated with a type of small vesicle (Pan and Johnstone, 1983; Pan and Johnstone, 1984); such extracellular vesicles (EVs) were defined as exosomes in 1989 (Johnstone et al., 1989). In the last decades, a series of EVs have been described, while the definition of EVs remains confusing among different reports (Hunter et al., 2008; Skog et al., 2008; Cocucci et al., 2009; Silva et al., 2011). Up to now, the different kinds of EVs are differentiated based on their size, content, and formation mechanisms; EVs mainly include apoptotic bodies, microvesicles, and exosomes (Al-Nedawi et al., 2009; Raposo and Stoorvogel, 2013), among which, apoptotic bodies and microvesicles are generated from plasma membrane, with a diameter of 800–5,000 nm and 200–1,000 nm, respectively (Camussi et al., 2011; Livshits et al., 2015), and exosomes are endogenous vesicles with a diameter of 30–150 nm (Mathivanan et al., 2010; Sampey et al., 2014; Zhang et al., 2019a). Since exosomes have been firstly discovered, they have been thought of as a cellular waste product, while in recent years, it has been found that tiny membrane vesicles contain cell-specific proteins, lipids, and nucleic acids, which can be delivered to other cells as signal molecules to change functions or other cells. These findings have sparked interest in cell secretory vesicles.

### The Formation of Exosomes

Exosomes, membrane-bound vesicles, with a diameter of 30–150 nm, are presented in nearly all kinds of biological fluids; the existence of exosomes has already been found in saliva, urine, semen, plasma, cerebral spinal fluid, bronchial fluid, serum, amniotic fluid, breast milk, bile, synovial fluid, tears, lymph, and gastric acid (Caby et al., 2005; Akers et al., 2013; Vojtech et al., 2014; Yoshida et al., 2014; Shi et al., 2015; Zlotogorski-Hurvitz et al., 2015; Milasan et al., 2016; Yoon and Chang, 2017; Li et al., 2018a; Dixon et al., 2018; Goto et al., 2018; Yuan et al., 2018). Exosomes are initially formed by endocytosis; the cell membrane is internalized to generate endosomes and then many small vesicles are formed inside the endosome by invaginating parts of the endosome membrane; such vesicles are called multivesicular bodies (MVBs). Ultimately, these MVBs fuse with the cell membrane, releasing the intraluminal endosomal vesicles into extracellular space by exocytosis to become exosomes (Heijnen et al., 1999; Gruenberg and van der Goot, 2006). They are also usually defined as intercellular communication vectors containing bioactive substances, including cytokines, proteins, lipids, mRNAs, miRNAs, non-coding RNAs, and ribosomal RNAs; the lipid bilayer of the exosomes protect them from ribonuclease degradation and help improve their biological efficiency in recipient cells.

### The Identification of Exosomes

Generally, the existence of exosomes is authenticated by a series of identifications to confirm; authentication methods vary from physical characteristics to surface molecular markers, including transmission electron microscopy (TEM), nanoparticle tracking analysis (NTA), and Western blot for molecular marker detection (Dragovic et al., 2011; Kowal et al., 2017; Jung and Mun, 2018). Through TEM detection, exosomes are visualized by electron microscopy after negative staining; exosomes usually appear as cup-shaped entities by transmission electron microscopy, but as hemisphere-shaped entities by cryoelectronic microscopy. There is a high degree of morphological diversity among exosomes isolated from different kinds of body fluids (Sahoo et al., 2011; Höög and Lötvall, 2015). Using the NTA method, the Brownian motion of individual vesicles is tracked, and their size and total concentrations are calculated using NTA software. NTA could measure cellular vesicles as small as 50 nm, which is far more sensitive than conventional flow cytometry (lower limit is 300 nm). Besides, their phenotype could be quickly determined by combining NTA with fluorescence measurement (Dragovic et al., 2011). From Western blotting analysis, exosome marker proteins include a family of four-transmembrane proteins, such as CD9, CD63, and CD81; cytoplasmic proteins like actin and annexins; and molecules involved in biological functions, including apoptotic transfer gene 2 interacting protein X (Alix), tumor susceptibility gene 101 protein (TSG101), heat shock protein (HSP70 and HSP90), and cell-secreted specific proteins, among which CD9, CD63, HSP70, and TSG101 are commonly used identification proteins for exosomes (Su et al., 2019).

### Communication of Exosomes

Cell–cell communication is an essential way to exchange information between cells, and exosomes play a pivotal role as vehicle for carrying information. When exosomes are secreted from host cells into recipient cells, they could regulate the biological activities of recipient cells by transferring proteins, nucleic acids, and lipids. Basically, exosome-mediated intercellular communications mainly occur in three mechanisms: First, the exosome membrane proteins could interact with the receptors, to activate intracellular signaling pathways of target cells (Munich et al., 2012). Second, in ECM, exosome membrane proteins could be cleaved by proteases, and the spliced fragments could act as ligands to bind to receptors on the cell membrane, thus activating intracellular signaling pathways (Tian et al., 2013). Third, exosome membranes directly fuse with recipient cell membranes, releasing their content such as proteins, mRNA, and microRNA into the cytosol (Mulcahy et al., 2014).

Compared to traditional gene therapy vectors, exosomes could protect and transfer bio factors as natural nanocarriers (van Dommelen et al., 2012). In the clinic, the applications of MSCs have been predominant due to their stronger functions than other cells, such as proliferation and differentiation ability *in vitro* (Yeo et al., 2013), and MSCs also generate a large amount of exosomes, which have low immunogenic properties. Several studies have shown that MSC exosomes may be more appropriate than MSCs in stem cell-based therapies (Yuan et al., 2020; Krut et al., 2021; Xing et al., 2021). Currently, numerous studies have reported the significance of exosomes in the treatment of IDD; the biological composition and functions of exosomes are based on different types of cells (Wang et al., 2021).

MicroRNAs (miRNAs) are a class of non-coding single-stranded RNA with a length of less than 22 nucleotides. Various studies have reported the associations between miRNA levels and IDD, including proliferation and apoptosis of NP cells, ECM regeneration, and inflammation response (Jing and Jiang, 2015; Li et al., 2015; Wang et al., 2015), and the dysregulated miRNA expression is widely observed in IDD (Zhao et al., 2014), which has essential roles in the progression of IDD, and it attracts much attention in delivering exosome-derived miRNAs in ameliorating IDD (Saravanan et al., 2019).

## Mechanisms of Exosomes in the Treatment of IDD

**FIGURE 1:**
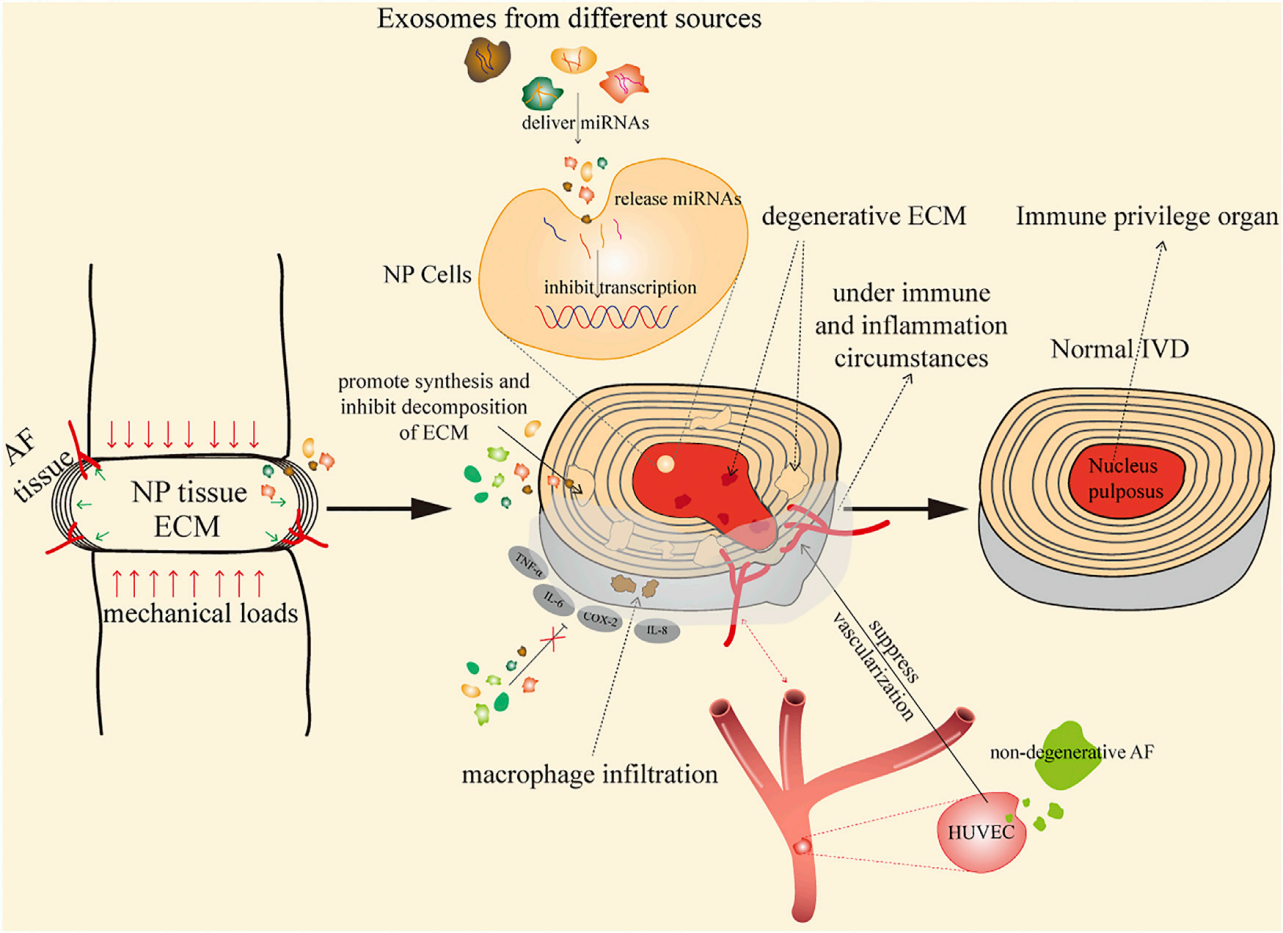
Mechanisms of exosomes in the treatment of IDD.

In disc degeneration, the main pathological changes are excessive degradation of ECM and reduction in AF and NP cells. Since the intervertebral disc is a closed, avascular structure, intervertebral injection is suggested to be an ideal method for the treatment of IDD (Hu et al., 2020a). Exosomes have been demonstrated to affect catabolism and anabolism of ECM by inhibiting MMPs, and most exosomes play roles in IDD mainly through releasing miRNAs (Zhang et al., 2019b); the internalization of PKH67 (most detected)-labeled exosomes into targeted cells indicates the involvement of exosomes in modulating these changes. Thus, exosome therapy is a promising therapeutic approach for IDD, which achieves its therapeutic effects through continuous release of miRNAs, proteins, and transcription factors that regulate metabolic disorders, microenvironment, and cell homeostasis (Wang et al., 2021). Existing studies have reported the application of different resources of exosomes in the treatment of IDD; here, their main therapeutic mechanisms are illustrated, as shown in Figure 1.

### By Improving Immune Microenvironment and Inflammation Reactions

As the largest avascular organ in the body, IVDs are located between vertebras, responsible for the sustainability, durability, and flexibility of the spine (Ji et al., 2018). NP cells are surrounded by AF and EPs, and they are trapped in the IVD since the formation of NP cells; this unique structure isolates NP tissues from the immune system of the host, and thus, IVDs are identified as an immune privileged organ (Sun et al., 2020a). Studies have found that various ingredients of NP induce auto-immune and inflammation responses after exposure to the host immune system during IDD (Capossela et al., 2014; Wang and Samartzis, 2014), and antigen–antibody complexes are commonly present in herniated NP tissue (Satoh et al., 1999).

The recruitment of immunocytes may lead to deterioration of IDD, through cell–cell communication and cytokine secretion. As for immunocyte types, activated T and B cells have been found to be elevated by autologous NP subcutaneously in a pig model (Geiss et al., 2007). Murai et al. (2010) have reported that macrophages and NK cells may recognize autologous NP cells and display positive cytotoxic effects, according to a comparison between wild-type mice and immune-deficient mice, and plasmacytoid dendritic cells, along with few macrophages and memory T cells, are also found in isolated and extruded discs (Geiss et al., 2014). Capossela et al. (2014) have also provided direct evidence of auto-immune response, showing that IgGs are found specific to collagen type I, II, and V and aggrecan in human degenerative IVD samples; these findings suggest that complicated immunocytes participate in auto-immune response of NP tissues in different stages. Moreover, inflammatory factors are also increased in the development of IDD. In an autograft model, Takada et al. (2012) have detected the high expression of TNF-α, IL-6, IL-8, cyclooxygenase 2, and macrophage infiltration. Consequently, these reports elucidate that with the damage of immune privilege in IVD, exposed NP tissues could promote auto-immune response, which finally leads to the activation of immunocytes as well as the infiltration of inflammatory factors. Previous research has also reported the association between autophagy and miRNAs, showing that dysregulation of the relationship between autophagy and miRNAs may accelerate the aging and apoptosis of NP cells (Akkoc and Gozuacik, 2020; Lan et al., 2021). NP cells firstly activate or repress the expression of specific miRNAs under stress condition, and then miRNAs regulate autophagy level by directly targeting ATG and signaling pathways to meet cellular demands (Zhou et al., 2016). More studies need to be further conducted, including the associations between exosomes and immunocytes, and whether these immunocytes could secrete exosomes and play a role in the treatment of IDD.

### By Suppressing Vascularization

Vascularization is widely observed in IDD, which is considered to play essential roles in the progression of IDD. Blood vessel invasion would cause activation of immunocytes and inflammatory factors, increase neuralization, and thereby damage the dynamic balance in IVD (Sun et al., 2020b). Existing studies have reported the pivotal roles of exosomes in inhibiting the growth of blood vessels; Cornejo et al. (2015) and Kwon et al. (2017) have found that soluble factors from notochordal cells could suppress endothelial cell invasion and vessel formation by inhibiting the VEGF signaling pathway; meanwhile, Sun et al. (2020b) have confirmed the finding that notochordal cell-derived exosomes could suppress proliferation of HUVECs; another research has also discovered the regulatory roles of AF exosomes, showing that degenerative AF exosomes promote migration of HUVECs and upregulate the expression of inflammatory factors (Sun et al., 2021). Furthermore, Liang et al. (2017) have reported that the co-culture system between PMSCs and endothelial progenitor cells (EPCs) enhances the angiogenic potential of EPCs through PDGF and the Notch signaling pathway (Komaki et al., 2017). Collectively, exosomes play an important role in the regulation of vascularization, different sources and cells may result in different outcomes, and detailed information about vascularization is fully discussed below.

### By Promoting Synthesis and Inhibiting Decomposition of ECM

Due to the special structure of AF that consists of 99% ECM and 1% AF cells, the ECM is of great significance to maintain the avascular structure and homeostasis of IVD. Focal proteoglycan loss has been noticed to cause alteration of ECM, which facilitates the growth of nerves and blood vessels (Stefanakis et al., 2012). Degenerative IVD possesses the common feature of significant loss of ECM, including COL2 and aggrecan. MMPs are chief catabolic factors responsible for these pathological changes, and the dysregulated expression of MMPs is widely observed and could enhance deterioration in IDD progression, including MMP-1, MMP-3, MMP-9, MMP-13, ADAMTS-4, and ADAMTS-5 (Zhang et al., 2021a). Exosomes have been demonstrated to affect the catabolism of ECM by inhibiting MMPs (Cabral et al., 2018; Zhang et al., 2019b). Almost all mechanisms ultimately promote regeneration of ECM and inhibit ECM degradation for IVD regeneration. Different nucleic acids transferred from exosomes exhibit their roles through multiple transductions, aiming at MMPs to modulate expression of ECM.

## Potential Sources of Applications by Exosomes During the Progression and Treatment of IDD

**FIGURE 2:**
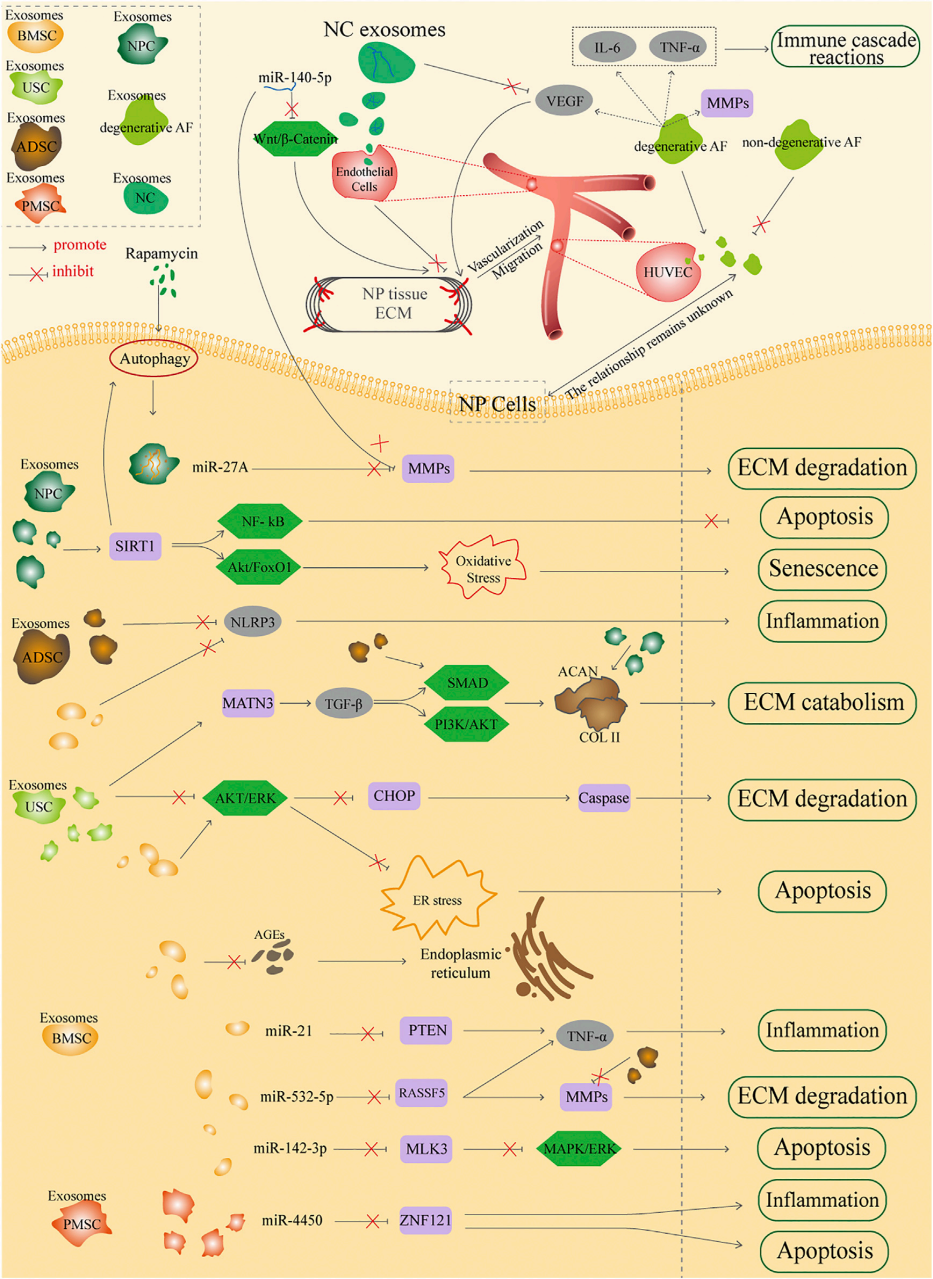
Potential sources of applications by exosomes in the treatment of IDD.

A number of exosomes from different sources have been reported, among which exosomes could be divided into two major parts based on their properties: stem cell-derived exosomes and non-stem cell-derived exosomes. They could be further classified according to source, such as BMSC, PMSC, USC, and ADSC exosomes from stem cell-derived exosomes, and AF, NP, and NC exosomes from non-stem cell-derived exosomes. This review aims to focus on the different sources of exosomes to elucidate the detailed mechanisms in the treatment of IDD; the whole mechanism is illustrated in Figure 2.

### Stem Cell-Derived Exosomes

As a worldwide issue, the treatment of IDD based on stem cells has been widely studied; the rationales of stem cell therapy are considered as replenishing disc cells through multipotent differentiation, promoting proliferation of NP cells, enhancing immune privilege, and reducing apoptosis and anti-inflammation (Ma et al., 2015). Studies have already confirmed the potential therapeutic efficacy of MSC transplantation in the treatment of IDD, and by inheriting the characteristics of MSCs, exosomes could also treat IDD through protecting NP cells from apoptosis, mitigating the inflammatory responses of disc, and promoting the synthesis of ECM (Lu et al., 2017; Xing et al., 2021).

The usage of exosomes as a cell-free product has continuously been regarded as substitute therapy against stem cell transplantation; the applications of exosomes rather than stem cells have advantages including low risk of tumorigenesis, malformations, and microinfarctions; convenience in collection and storage; increase of sustained biological activity; stability; and minimal immunogenicity when transplantation (Tao et al., 2018). Exosomes have been demonstrated to affect the catabolism of ECM by inhibiting MMPs (Cabral et al., 2018; Zhang et al., 2019b). Consequently, exosome therapy is a novel promising therapeutic approach for IDD; the efficacies are achieved from the continuous release of microRNAs, proteins, and transcriptome factors to regulate metabolic disorders, microenvironment, and cell homeostasis (Xing et al., 2021). Different stem cell-derived exosomes are highly specific in treating IDD; compared to traditional gene therapy vector, exosomes could be considered as nanocarriers to transfer specific molecules including miRNA, siRNA, or AntagomiR to recipient cells through endocytosis and membrane fusion (Zhou et al., 2016; van den Boorn et al., 2011). The detailed mechanisms of MSC exosomes in the treatment of IDD are discussed as follows:

#### Exosomes Derived From Bone Marrow Mesenchymal Stem Cells

BMSCs derived from mesoderm are a type of stem cell with multidirectional differentiation potential. BMSC transplantation, which serves as a representative cell therapy, is becoming prevalent in the field of bone regeneration, cartilage repair, spinal cord injury, and IDD (Sakai and Andersson, 2015; Hejazi et al., 2021; Jiang et al., 2021; Maldonado-Lasunción et al., 2021). However, stem cell therapy still has many limitations such as difficulty in obtaining cells, susceptibility to aging, potential tumorigenesis, and immune rejection, which greatly limit the application in the field of regenerative medicine (Dou et al., 2021).

BMSC exosomes have advantages of low immunogenicity and suitability for the IVD microenvironment, and it could provide cell-free therapy instead of the traditional BMSC therapy. BMSC exosomes have similar functions to BMSC, such as restoring tissue damage, inhibiting inflammation, and regulating immune microenvironment. Existing research has reported that BMSC exosomes could modulate the degenerative NP gene toward a healthy NP gene phenotype (Lu et al., 2017). High levels of inflammatory factors, like TNF-α, have been demonstrated to result in excessive apoptosis of NP cells and cause IDD progression (Risbud and Shapiro, 2014), and growing lines of evidence have indicated the roles of miRNAs in the progression of IDD, showing that exosomes transport these miRNAs into IVD (Lan et al., 2016). Cheng et al. (2018) showed that in an IDD rat model, BMSC exosomes inhibited TNF-α-induced NP cell apoptosis, and further analysis suggested that exosomes were rich in miR-21 and inhibited NP cell apoptosis by specifically targeting PTEN through transporting miR-21 into NP cells. In a study of degenerated and normal NP cells, Xia et al. (2019) have found various proteins related to inflammatory responses in IVD; the exosomes suppress the progression of IDD by targeting the activation of inflammatory mediators NLRP3 in NP cells, which is an effective therapeutic target of IDD. Zhu et al. (2020a) have suggested highly expressed miR-532-5p in BMSC exosomes, which could suppress the TNF-α-induced apoptosis, ECM degradation, and fibrosis deposition in NP cells by delivering miR-532-5p *via* targeting RASSF5. RASSF5, a major member of RASSF5 family, could selectively trigger RAS and function as a tumor suppressor, which is highly correlated with cell proliferation, apoptosis, and tumorigenicity among many neoplasms (Antoniou et al., 1996; Suryaraja et al., 2013). MMP-13, a regulatory gene of ECM degradation in IDD, has also been proven to be complementary with miR-532-5p (Mohanakrishnan et al., 2018). They revealed two therapeutic targets to attenuate IDD: miR-532-5p in BMSC exosomes could decrease the apoptosis of NP cells by targeting RASSF5 and inhibit ECM degradation by targeting MMP-13 in NP cells.

Autophagy is tightly connected to aging as well as apoptosis in the pathogenesis of disorders, including cancer, osteoarthritis, and degenerative diseases. Autophagy exists in degenerative disc and is an effective approach in regulating ECM metabolism in IVD (Zhang et al., 2021b). Related studies have elucidated that BMSC exosomes promote proliferation of AF cells by inhibiting expression of inflammatory cytokines stimulated by IL-1β. The PI3K/AKT/mTOR signaling pathway is activated by BMSC exosomes, which is an essential signaling pathway regulating autophagy (Li et al., 2020), and a previous study has also confirmed the regulation of exosomes in the PI3K/AKT/mTOR signaling pathway and that human umbilical cord mesenchymal stem cell-derived exosomes inhibited apoptosis of H9C2 cells *via* autophagy regulation (Liu et al., 2019).

Unfolded protein response (UPR) is an evolutionarily conservative reaction induced by endoplasmic reticulum (ER) stress; it is the most typical response among ER stress. When any abnormal reaction of ER occurs, ER stress could be triggered to protect the homeostasis of ER (Hetz, 2012). However, severe or prolonged ER stress could hyperactivate UPR, which results in excessive degradation of cellular proteins, and eventually leads to cell demise (Kim et al., 2008). AGEs (advanced glycation end products), also called the Millard reaction, are non-reducible substances generated by the polymerization of sugars and proteins through a series of reactions (Chan et al., 2016). AGEs commonly accumulate in aging and degenerative diseases, and are closely related to inflammation, metabolic dysfunction, and ER stress (Chiang et al., 2016). Both AGEs and ER stress are highly correlated with IDD; higher levels of AGEs are detected in degenerative disc, and AGEs are also connected to Pfirrmann grades of IVD (Fields et al., 2015; Song et al., 2018). Liao et al. (2019) have demonstrated that according to an *in vitro* and rat model, BMSC exosomes reduce AGE-induced ER stress, inhibit the activation of UPR, and ameliorate NP cell apoptosis. AGE accumulations are reported to induce ER stress, leading to cell apoptosis by induction of prolonged UPR, and the accumulation of CHOP could promote the cleavage of caspase-3 and caspase-12, resulting in cell apoptosis (Adamopoulos et al., 2016; Go et al., 2017). Through detecting the related signaling pathway AKT, ERK, and ER-related CHOP protein, they elucidated that BMSC exosomes significantly activate AKT and ERK signaling, which reduced expression of CHOP, then further inhibited caspase-3 and caspase-12, and ultimately decreased the apoptosis of NP cells and catabolism of ECM, thus preventing IDD.

The mitogen-activated protein kinase (MAPK) family consists of ERK1/2, JNK, and p38 MAPK proteins, and studies show that ERK is mediated to cell proliferation and inflammation (Sawe et al., 2008); p38 MAPKs are a type of proinflammatory mediator, and p38 MAPKs and JNK both modulate cell apoptosis and inflammation (Yang et al., 2016; Lai et al., 2019). Increasing lines of evidence have shown that the activation of MAPK transduction is activated and tightly correlated to ECM degradation, cell aging, apoptosis, and inflammatory reactions in IDD (Zhang et al., 2021b). MLK3 belongs to serine/threonine MAPK kinase (MAP3K) and is aberrantly expressed in mammals, which mediates cell migration and invasion in various diseases (Zhang et al., 2014a; Lan et al., 2017; Misek et al., 2017). Zhu et al. (2020b) have revealed the relationships between miR-142-3p and IDD, that miR-142-3p is overexpressed in BMSC exosomes and miR-142-3p excreted from BMSC exosomes could ameliorate NP cell injury *via* targeting MLK3, which further inhibits the activation of the MAPK signaling pathway. They have proved that exosomal miR-142-3p and the subsequent MLK3/MAPK cascade transduction could be as an effective approach in attenuating the progression of IDD.

#### Exosomes From Urine-Derived Stem Cells

Stem cells are regarded as ideal cells for IVD degeneration, since they could prevent IVD tissues from aging and apoptosis. The applications of exosomes from BMSC are widely used in various diseases including IDD, while the limited sources of BMSC, expensive costs to obtain BMSC, and the physical trauma in the acquisition process (Sakai and Andersson, 2015) prompt researchers to find safer, less expensive, and more effective exosomes from other sources. Studies have discovered that a subpopulation of cells isolated from urine have similar characteristics to BMSCs, such as multidirectional differentiation capacity, clonogenicity, expansion patterns, self-renewal capacity, and paracrine properties (Bodin et al., 2010; Wu et al., 2011a; Bharadwaj et al., 2011; Wu et al., 2011b). These cells are thereby named urine-derived cells or USCs. Compared to BMSCs, USCs possess several merits: first, they could be collected through a simple, safe, low-cost, and non-invasive manner; second, the acquisition procedures do not violate ethics; last, USCs have a low cost of culture and a faster proliferative rate; besides, they could avoid immunological rejection when using autologous therapy (Qin et al., 2014; Pavathuparambil Abdul Manaph et al., 2018). Therefore, USCs, as a novel cell source, may provide a more effective way in the amelioration of IDD.

IVD tissues are subjected to different levels of mechanical stress in daily work and life, which have essential roles in spinal biomechanics. Wang et al. (2013) and Gawri et al. (2014) have discovered that excessive mechanical stress induces cell apoptosis, promotes ECM-degrading enzymes, and finally leads to IDD. As mentioned above, certain levels of UPR in ER stress could prevent cells from external stimulus and restore cell homeostasis. Proper biomechanical loads play pivotal roles in the structure and function of articular cartilage, while abnormal and continuous biomechanical stimulation could lead to accumulation of misfolded proteins in ER lumen, resulting in continuous ER stress and then cell apoptosis (Wang and Kaufman, 2016; Hunt et al., 2020; Yang et al., 2020). Xiang et al. (2020) have discovered the therapeutic application of USC exosomes in IDD under high mechanical loads. They have elucidated that USC exosomes suppress excessive activation of UPR, cell apoptosis, and disc degeneration through AKT and ERK signaling pathways, which is consistent with the above finding that AKT/ERK transduction is activated in BMSC exosomes and behave in AGE-induced ER stress (Liao et al., 2019). These findings imply that AKT/ERK signaling pathways mediated by exosomes play active roles in AGE-induced or biomechanical load-induced IDD, leading to caspase reduction by inhibiting CHOP protein, and thereby reducing ECM degradation and NP cell apoptosis. Consequently, inhibiting ER stress induced by AGEs *via* BMSC or USC exosomes may be a potential therapeutic target in the treatment of IDD. However, the type of nucleic acids exosomes carry to mediate the AKT/ERK signaling pathway still needs to be further analyzed.

COL2 and proteoglycan (chiefly aggrecan, namely, ACAN) are considered as crucial ECMs for discs to maintain their proper functions, especially for NP cells (Vo et al., 2013). Guo et al. (2021) have suggested that the methods of rebalancing disordered COL2 and ACAN and increasing the synthesis is regarded as a major point for ameliorating IDD progression. They have found that USC exosomes could induce cell proliferation of NP cells and ECM synthesis. TGF-β is a multifunctional cytokine, which modulates cell fate and plasticity, and MATN3 could be directly bound to specific integrins, promoting dissociation and activation of TGF-β and affecting downstream gene activation (Pullig et al., 2002). Lines of evidence have demonstrated that multiple cellular reactions induced by TGF-β are modulated *via* a canonical SMAD pathway and noncanonical pathways like PI3K/AKT transduction (Goc et al., 2011; Hamidi et al., 2017). Guo et al. (2021) have confirmed that USC exosomes are found to be rich in MATN3 protein, and exosomal MATN3 could ameliorate IDD progression by activating the TGF-β/SMAD pathway to promote the expression of COL2 and ACAN in ECM and NP cells, and by triggering the TGF-β/PI3K/AKT pathway to function in cell proliferation and antisenescence (Feng and Qiu, 2018).

#### Exosomes Derived From Human Placental Mesenchymal Stem Cells

As mentioned above, although BMSCs are regarded as a gold standard among other MSCs, the difficulty and efficacies in obtaining BMSCs have forced scientists to find novel and multifunctional sources. Among them, the placenta has attracted much attention as an alternative source of BMSCs, due to its abundance and ease of availability (Mathew et al., 2020). It has been reported that a large amount of PMSC could be collected from a small tissue chunk (In 't Anker et al., 2004; Parolini et al., 2008), making the acquisition of PMSC easier, together with its exosomes. As a transient materno-fetal organ, the placenta is disposed of after delivery and involves non-invasive procedures, making it a more convenient source (Mathew et al., 2020; Gorodetsky and Aicher, 2021). The applications of PMSC exosomes have been reported to affect osteogenic and adipogenic differentiation by regulating OCT4 and NANOG in dermal fibroblasts (Tooi et al., 2016). The main advantage of PMSC exosome-based therapies appears to be the secretion of a wide range of anti-inflammatory and pro-regenerative factors, which makes it possible for PMSC exosomes to treat IDD.

Yuan et al. (2020) have discovered the applications of PMSC exosomes in IDD; they have found that miR-4450 is highly expressed in degeneration disc and PMSC exosomes have therapeutic effects on NP cells, and by inhibiting miR-4450 and thus upregulating downstream gene ZNF121 to alleviate apoptosis, inflammation, and necrosis of NP cells, it could even improve gait abnormality *in vivo* caused by IDD. ZNF121 has been demonstrated to be the target of miR-4450; as one of the biggest families of regulatory proteins in human cells, ZNF121 plays an essential role in the development and differentiation of various diseases (Ladomery and Dellaire, 2002). It is modulated by multi-miRNAs and causes diseases including breast cancer, cell proliferation of childhood neuroblastoma (miR-1427), and invasion of gastric cancer (miR-204-5p) (Luo et al., 2016; Wu et al., 2018; Huan et al., 2019). In addition to finding that PMSC exosomes could directly inhibit miR-4450 and then upregulate ZNF121, and thus ameliorate the apoptosis of NP cells and IDD progression, Yuan et al. also successfully delivered AntagomiR-4450 into exosomes, which made it more effective than exosomes, further verifying the efficacy of PMSC exosomes in treating IDD alone and their natural merits as an oligonucleotide carrier. The method of using the direct role of siRNA has been considered as an excellent therapeutic approach, while its low bioavailability limits the full realization of its clinical potential (El Andaloussi et al., 2013). These findings imply that the containing of oligonucleotide-based vesicles or drugs opens a novel avenue for target therapy and that PMSC exosomes could be regarded as nanocarriers to deliver specific molecules, like siRNA or AntagomiR, and then transport the contents to target cells through membrane fusion or endocytosis (Zhou et al., 2016; Yuan et al., 2020).

Although PMSC exosomes could be regarded as a vector that delivers miRNAs, its vascularization characteristic is not suitable for treating IDD, due to the avascular structure of IVD. The detailed mechanisms between neovascularization and exosomes would be discussed later.

#### Exosomes From Adipose-Derived Mesenchymal Stem Cells

Like USCs and PMSCs, ADSCs also come from a wide range of sources and the damage to people is negligible when collected from the human body (Xing et al., 2021); besides, the applications of ADSC exosomes have been confirmed to accelerate the proliferation and inhibit apoptosis of target cells, and they have anti-inflammatory effects in the treatment of ruptured tendon (Shi et al., 2018; Zhang et al., 2021c), which suggests their potential ability in degenerative diseases like IDD. Studies have obtained exosomes from ADSC immobilized on ECM hydrogels and then successfully construct an injectable thermosensitive hydrogel system through mutual crossing of ADSC exosomes and ECM hydrogels to restore the microenvironment as well as pyroptosis of IDD (Xing et al., 2021). They have confirmed that ADSC exosomes could regulate the expression of MMPs directly, inhibit the catabolism of ECM, and thus promote the accumulation of aggrecan and COL2. Besides, ADSC exosomes could also inactivate the inflammatory factor NLRP3, retard their release, and then affect the pyroptosis and survival of NP cells. Compared to traditional exosome release, dECM (decellularization ECM) exosome combination possesses several merits, such as preventing immune response, high load rate, slow release of exosomes, and prolonging their activation time. The researchers established dECM to load engineered exosomes to delivery drugs into IVD and NP cells, and eventually enhance the drug effects of small molecules.

Besides, ADSC exosomes have also been found to activate the SMAD signaling pathway in tendon healing (Liu et al., 2021a), which is consistent with the above findings that SMAD transduction is upregulated by BMSC and USC exosomes. Thus, further analysis should be expanded about the SMAD signaling pathway *via* ADSC exosomes in the treatment of IDD, and the correlations between SMAD and other different sources of exosomes should also be further observed.

### Non-Stem Cell-Derived Exosomes

In addition to the advantages of stem cell-derived exosomes mentioned above, they also possess disadvantages: they may cause hypertrophic differentiation of newly formed tissues, together with unexpected angiogenesis (Chen et al., 2018), which are the limitations in such closed and avascular IVD. However, human cartilage cell-derived exosomes have been found to facilitate chondrogenesis of cartilage progenitor cells without such deficiency (Chen et al., 2018), and normal chondrocytes are located in a similar avascular and closed environment and share many common features like NP cells (Rosenthal et al., 2015). Additionally, existing studies have reported that exosomes from non-stem cells like NP cells could promote the migration of MSC cells and induce MSC into IVD differentiation (NP-like phenotype) (Chen et al., 2018); AF-derived exosomes could also have the same functions. At present, non-stem cell-derived exosomes that could ameliorate IDD progression are from NP, AF, and notochordal cells; the specific mechanisms of these exosomes are discussed as follows:

#### Exosomes Derived From Nucleus Pulposus

Recent studies of exosomes regarding IDD mainly focus on MSC-derived exosomes, while the application of NP cells has not been widely reported yet. Zhang et al. (2021a) have discovered that NP cells could secrete exosomes and deliver miR-27A to prevent ECM degradation by targeting MMP-13. Autophagy activation could promote release of NP exosomes and thereby prevent the NP cell matrix from degradation, and it also ameliorates degeneration of IVD, at least partly *via* exosomal miR-27A, which targets and inhibits MMP-13 expression. In an *in vitro* mutual experiment, Lu et al. (2017) have elucidated the interaction roles between NP and BMSC cells *via* exosomes, that BMSCs could spontaneously migrate across the transwell membrane to IVD in a dose-dependent manner induced by NP exosomes, and NP exosomes could induce BMSC differentiation toward NP-like lineage, suggesting that NP exosomes could recruit BMSCs and induce BMSC multidirectional differentiation, and then replenish IVD cells and appropriate ECM. Besides, they have demonstrated that NP exosomes are more effective in inducing BMSCs to differentiate toward NP-like cells than an indirect coculture system of BMSCs and NP cells. Strassburg et al. (2012) also reported that the formation of gap junctions and cell fusion are not the predominant mechanisms of interaction; intercellular transfer of membrane components is the main interactive mechanism between BMSCs and NP cells. These findings indicate that direct injection of BMSCs into IVD is not effective compared to injection of NP exosomes into IVD, which is consistent with the finding of Sun et al. (2021) that indirect cell–cell communication and paracrine are predominant in keeping the avascular condition of IVD. However, the detailed interactive mechanisms between BMSC and NP cells remain unclear, and more specific components like proteins, miRNAs, transcription factors, and lipids are expected for further research. Furthermore, interactions between NP cells or NP exosomes and other cells in IVD (such as AF cells and cartilage EP cells) should be further analyzed (Hampton et al., 1989) to reveal their roles in the progression of IDD.

Continuing with autophagy mentioned above, autophagy is considered as a protective procedure for cell survival under stress situations; in such process, excessive proteins or aging organelles in cells are degraded to provide extra energies and, in most cases, restore homeostasis (Mizushima, 2009). Highly conservative serine/threonine kinase, as a mechanistic target of rapamycin (mTOR, also known as mammalian target of rapamycin), is a crucial cell growth regulatory cytokine that connects cellular metabolism and growth with multiple environment inputs (Kim and Guan, 2019). mTOR as a core component autophagic pathway could negatively regulate autophagy. Several studies have implied that autophagy activation could maintain the balance of NPC, ECM, and vitality under inflammation (Jiang et al., 2013; Li et al., 2019). Serving as an effective agonist of autophagy and immunosuppressor, the application of rapamycin has been demonstrated to increase the release of NP exosomes, upregulate expression of miR-27A, and target MMP-13 to prevent ECM degradation. Rapamycin may also overcome the limitation that non-stem cell exosomes are difficult to collect and reproduce (Zhang et al., 2021a). Moreover, NP exosomes have been found to be secreted in an autophagy-dependent manner, and rapamycin has been confirmed to stimulate NP cell release exosomes *via* the RhoC/ROCK2 signaling pathway (Hu et al., 2020b). Consequently, these findings prove that NP exosomes also have high therapeutic potentials and provide us with novel insights into the usage of NP exosomes *via* rapamycin activation in the treatment of IDD.

NP cells also secrete different numbers of exosomes in different degenerative grades; exosomes secreted from high degenerative levels of NP cells could promote the apoptosis and inhibit the proliferation of NP cells (Song et al., 2020), indicating a positive correlation between the functions of degenerative exosomes and IDD progression, as well as the duality of exosome application. TGF-β is abundant in NP exosomes, which is well studied to transform BMSCs into NP-like cells (Steck et al., 2005), suggesting the direct functions of NP exosomes to BMSCs and the ability to carry specific genes through NP exosomes. Circular RNAs (circRNAs) are a class of covalently closed non-coding RNA molecules generated by reverse splicing of the exon of precursor mRNA in eukaryotes (Li et al., 2018b); studies have shown the tight connections between circRNAs and IDD (Wang et al., 2018). A rat model in Song et al. (2020) has suggested that circRNA_0000253 is highly expressed in IDD rat with positive correlations; they could target and absorb miRNA141-5p, in a negative manner with IDD, and thus downregulate the expression of Sirt1. Since the continuous release of exosomes, the degenerative exosomes from NP cells may secrete and further affect neighboring normal tissues and thus aggravate the progression of IDD. As a result, there is an urgent need to discover new approaches targeting degenerative NP cells, among which circRNA_0000253 and miRNA141-5p have been demonstrated to be effective, through delivering siRNA-circRNA_0000253 or mimic-miRNA141-5p into degenerative NP cells to prevent ECM degradation. Related signaling pathways are circRNA_0000253/miRNA141-5p/Sirt1. Given the multiple functions and multiple advantages of exosomes, exosomes alone or with specific genes or drugs would be a suitable choice for cell-free strategies in the treatment of IDD.

Sirt1 (Sirtuin 1) is a nicotinamide adenine dinucleotide (NAD+)-dependent histone deacetylase, which could reduce apoptosis in different cells and correlated with various diseases including cancer, metabolic disorders, COPD, and aging-related disorders like degenerative and cardiovascular diseases (Dai et al., 2018). Sirt1 participates in many pivotal cellular biological processes, such as inflammatory response, oxidative stress, and mitochondrial functional homeostasis, which are consistent with the mechanisms in IDD progression in that Sirt1 plays a protective role in senescence and apoptosis of NP cells (Guo et al., 2017; Zhang et al., 2019c). Guo et al. (2017) have found that Pfirrmann grade is negatively correlated with Sirt1 expression in IDD. *In vitro* experiments have verified that resveratrol and quercetin could promote cell proliferation and senescence-related protein expression (Guo et al., 2017; Wang et al., 2020b). Shen et al. (2016) and He et al. (2019) have shown that Sirt1 prevents NP cells from apoptosis *via* TLR2/Sirt1/NF-kB transduction, and Sirt1 could ameliorate oxidative stress-induced senescence of NP cells regulated by the Akt/FoxO1 pathway; a previous study has also reported that Sirt1 could modulate autophagy, which may further mediate the status of IVD (Wang et al., 2020b). These observations imply that Sirt1 could serve as a novel therapeutic target in the treatment of IDD, and the applications of exosomes mediating Sirt1 have already been demonstrated in some diseases like brain injury, photodamage, and neuronal autophagy (Chen et al., 2020; Liu et al., 2021b; Wu et al., 2021). To the best of our knowledge, the applications of Sirt1 *via* exosomes in the treatment of IDD have not been reported; further relevant studies could focus on exosomes and Sirt1 to reveal detailed mechanisms.

In terms of osteoarthritic cartilage, osteoarthritic cartilage and degenerative IVD possess the common feature of significant loss of ECM, including COL2 and aggrecan; MMPs are chief catabolic factors responsible for these pathological changes. Recent studies have discovered that the applications of autophagy agonists like rapamycin, chloramphenicol, and ozone inhibit MMP-13 expression and thus attenuate OA processes (Zhao et al., 2018; Ma et al., 2019; Wu et al., 2019). A previous study has reported the association between autophagy and exosome release in chondrocytes, that they depend on caspase-3 and Rho/ROCK transduction (Rosenthal et al., 2015); these findings are consistent with the autophagy-activated signaling pathway of exosomes secreted from NP cells mentioned above. From inverted phase contrast microscopy, NP cells are presented as polygonal shape, and toluidine blue staining suggests that there are a large number of notochord cells in IVD. Further IHC analysis also shows the abundance of COL2; these results all confirm that NP cells possess cartilage-like characteristics (Zhang et al., 2021a). These results elucidate the tight connections between chondrocytes and NP cells, based on the similar features of chondrocytes and NP cells; the wide research about chondrocytes and subsequent research regarding NP cells could focus on the interactions with chondrocytes, to find novel ideas for treating IDD.

#### Exosomes Derived From Annulus Fibrosis

AF, the cells enclosing NP, consists of concentric layers composed of alternatively aligned oblique collagen fibers (mainly type I collagen) interspersed with AF cells (Kadow et al., 2015), which serve as a physical barrier to separate the internal NP and external blood vessels and immune system. Due to the special structure of AF that consists of 99% ECM and 1% AF cells, the indirect cell–cell contact or paracrine of AF cells may play pivotal roles in maintaining avascular conditions in IVD. Vascularization has been widely observed in IDD and has been considered as a pathological feature, which mainly occurs in AF tissue, and blood vessels grow and infiltrate inwards, could cause NP tissue exposure to immune system, and thereby damage immune privilege of IVD (Kim et al., 1981; Di Martino et al., 2013). Sun et al. (2021) have verified that the exosomes could be released by both degenerated and non-degenerated AF cells; they could also be internalized by HUVECs, and degenerative AF exosomes could enforce HUVEC migration faster than non-degenerative AF exosomes, elucidating that degenerative AF cells could induce IVD vascularization *via* exosome-mediated effects, while non-degenerative AF tissues could suppress blood vessels ingrowth as a physical barrier, and its exosomes could also be regarded as an angiogenesis inhibitor to maintain the healthy avascular condition of IVD. These results imply that AF exosomes could serve as one of the mediators in intercellular communication of IVD and modulate the vascularization of IVD.

Within the region of IVD, the position of AF and blood vessels is very close to each other, which may hypothesize the interactive potential effects between AF and vascular endothelial cells. Pohl et al. (2016) have discovered that endothelial microparticles secreted from vascular endothelial cells enhance MMP expression in AF cells, suggesting that the vascular endothelial cells could act on AF cells through microparticle delivery, and it could help blood vessel ingrowth into IVD by promoting ECM catabolism. Together with the findings that degenerative AF cells affect vascularization of vascular endothelial cells by delivering AF exosomes (Sun et al., 2021). Both AF and vascular endothelial cells secrete microparticles like exosomes, and they endocytose microparticles secreted from each other, and these reports all display the essential roles of microparticles/exosomes in AF and blood vessel communication. Besides, degenerative AF exosomes could lead to upregulation of IL-6, TNF-α, MMP-3, and MMP-13 as well as VEGF, which are consistent with previous findings that these factors could cause NP cell apoptosis and IDD progression, and these indicators could induce the inflammation of IVD. VEGF is a major proangiogenic factor that could trigger the growth, expansion, and relocation of endothelial cells, and play essential roles in vascularization of IVD (Capossela et al., 2018). These findings suggest the roles of degenerative AF exosomes; if situations are not promptly treated and intervened, IVD progression would produce a vicious cycle by degenerative AF exosomes, leading to further development of IDD.

Up to now, the existence and functions of AF exosomes have been rarely reported; although the interactions between AF exosomes and endothelial cell HUVECs have been verified, the relevant bioactive substances in AF exosomes related to these effects have not been discovered, and more research should be conducted to explore the potential molecular mechanisms and signaling pathways. Besides, only one kind of vascular-related cells—HUVECs—have been conducted to analyze the interactions with AF exosomes, while other vascular cells, like vascular smooth muscle cells, or arterial endothelial cells, have not been fully understood. Furthermore, due to the special position of AF and NP cells, whether AF have interactions with NP *via* exosomes still need to be further analyzed. Consequently, the application of AF exosomes is a promising research orientation whether in IDD or other fields, and these issues need to be followed up and resolved by researchers.

#### Exosomes Derived From Notochordal Cells

Notochord is an embryonic structure of chordates; during the embryogenesis period, the rod-shaped notochord is enclosed from the vertebral body and develops to the NP tissue soon in the early stage of fetal life (Cornejo et al., 2015), and blood vessels recede and vanish slowly from IVD. In human IVD of immature individuals (embryonic, fetal, and juvenile), NP tissues are mainly populated by large vacuolated notochordal cells, while IVDs in adult are populated with small and non-vacuolated chondrocyte-like cells (Bach et al., 2018), and with the aging of humans, early IDD begins to happen with the disappearance of NC cells (Risbud and Shapiro, 2011). Several *in vivo* experiments have verified the important roles in maintaining homeostasis of IVD (Bergknut et al., 2013) and that NC loss coincides with the onset of IDD. Increasing lines of evidence have also concluded the roles of NC in the development and functions of IVD: NC could stimulate proliferation of degenerative NP cells as well as BMSCs (de Vries et al., 2015; Bai et al., 2017), and it may stimulate chondrogenic differentiation, reducing natural cell necrosis. It has also been demonstrated to inhibit angiogenesis and maintain the avascular state of human IVD (Gruber et al., 2006; Mehrkens et al., 2017). In conclusion, the functions of NC indicate the essential effects and the potential roles in maintaining the NP tissues and IVD healthy, and therefore, NC could be regarded as a promising source for regenerative and symptom amelioration for IVD disease.

The existence of NC exosomes has firstly been confirmed by Sun et al. (2020b); they have found the best appropriate mechanical stress to stimulate the release of NC exosomes, and that NC exosomes could be internalized by endothelial cells. Mechanical stress is of great importance considering the environment of IVD, which is implicated as the predominant inductive cause of IDD; they are interconnected and amplify each other, and cellular physiology is strongly affected by mechanical loading (Vergroesen et al., 2015). In the early stage of spine development, NC cells are squeezed into IVD through the thrust of forming vertebrae, and then they enter the specific physiological environment and are compressed by mechanical force. Compressive load cultures have been found to induce the CK8 phosphorylation and downregulation in NP cells (Sun et al., 2013a), and NC cells are more resistant to mechanical forces compared to NP cells (Saggese et al., 2020), while high mechanical stress may lead to the exhaustion of NC resources (Hong et al., 2018). These findings hypothesize that the appropriate range of mechanical force is necessary to maintain the normal survival and function of NC cells, as well as the secretion of exosomes; 0.5 Mpa is found to be the suitable mechanical condition for inducing secretion of NC exosomes and modulating related functions.

NC exosomes have also been unveiled to deliver miR-140-5p to endothelial cells and then inhibit angiogenesis to maintain the avascular status of IVD, which are achieved by the Wnt/β-catenin signaling pathway (Sun et al., 2020b). Among them, miR-140-5p has been proven to participate in cell migration, proliferation, and metastasis (Rothman et al., 2016; Fang et al., 2017), and the exosomal miR-140-5p from NC could suppress the expression of MMP-2 and MMP-7 and thus ameliorate NP cell apoptosis and IDD. Wnt11, one of Wnt family members, has been shown to stimulate the proliferation, migration, and invasion of various types of cells *via* inducing β-catenin (Stefater et al., 2011; Franco et al., 2016), which is regarded as the pivotal downstream transduction of Wnt; these are consistent with the interactions *via* NC exosomes, that Wnt transduction is involved in angiogenesis through the modulation of endothelial cell proliferation and vascular sprouting (Zhang et al., 2017). Consequently, NC may have anti-angiogenesis ability *via* the NC-exosomal miR-140-5p/Wnt/β-catenin axis. According to their mass spectrometry study, Matta et al. (2017) have discovered from a non-chondrodystrophic dog notochordal cell conditioned medium that transforming growth factor beta1 (TGF-β1) and connective tissue growth (CTGF) are major hubs in protein interaction networks, which are essential for the homeostasis regulation of healthy NP tissues. Interestingly, VEGFA is also predicted to be inhibited among growth factors in NC cells (Rodrigues-Pinto et al., 2018). The identification of key biological factors derived from NC cells that delay the progression of IDD is still at an early stage; based on these findings, more studies about the functions mediated by NC exosomes need further exploration.

## The Relationships Between IVD Vascularization, Inflammation and IVD Cells, and Exosomes

As the largest avascular organ, IVDs are composed of three parts, namely, central NP cells, surrounding AF cells, and the adjacent cartilage endplates. Generally, blood vessels are confined to the outer surface of AF cells in normal IVD, while neovascularization has been widely observed and considered as a common pathological phenomenon in IDD. The inward growth of blood vessels causes NP exposure to immune system, leading to permeation of inflammatory factors and immune cells into NP tissues, and finally damage to the immune privilege and homeostasis of IVD (Sun et al., 2021). Additionally, the ingrowth of blood vessels in IVD enhances neuralization and pain sensitization (Choi, 2009); it also affects the phenotype and functions of NP cells by upregulating oxygen concentration in NP tissue (Wang et al., 2019). Therefore, the maintenance of the avascular condition of IVD is essential to sustain the homeostasis and functions of normal IVD (Chu et al., 2018).

In terms of the mechanisms of vascularization in IDD, it is generally considered to be the breakdown of physical barrier, such as the fissure of AF tissue, together with the increased expression of pro-angiogenesis factors, like VEGF and platelet-derived growth factor (PDGF) (Tolonen et al., 1997; Fujita et al., 2008), and pro-inflammatory cytokines like IL-1β and TNF-α (Lee et al., 2011; Risbud and Shapiro, 2014), that finally induce invasion of blood vessels. Focal proteoglycan loss has been noticed to cause alteration of ECM, which facilitates the growth of nerves and blood vessels (Stefanakis et al., 2012); increased blood vessel ingrowth is correlated with proteoglycan depletion AF lesion (Moon et al., 2012) and IVD aggrecan could inhibit migration and invasion of endothelial cells (Johnson et al., 2005). Furthermore, mechanical loading has also been proven to influence the capacity of IVD to stimulate the migration of endothelial cells (Neidlinger-Wilke et al., 2009). These results demonstrate the pivotal roles of a passive physical barrier in preventing IVD angiogenesis. As for molecular level, Wiet et al. (2017) have described the interactions between mast cells and IVD: that healthy AF culture medium could suppress the activation of mast cells by downregulating expression of inflammatory cytokines and then inhibit mast cell-induced angiogenesis. Cornejo et al. (2015) have provided evidence that soluble factors derived from notochordal-rich IVD could suppress angiogenesis *via* inhibiting VEGF signaling pathways, and NC-derived ligands are of significance in targeting neurovascular ingrowth and pain in the degenerative IVD. A previous study has also reported the importance of Fas–FasL (Fas Ligand) interaction, showing that FasL could induce apoptosis of endothelial cells (Sun et al., 2013b); besides, FasL generated by IVD could mediate the apoptosis of Fas-bearing cancer cells (Park et al., 2007), and the Fas–FasL network may provide a novel target for the treatment strategies of IDD. These results all suggest that these factors or cytokines might be the molecular monitor for maintaining functions through inducing apoptosis of vascular endothelial cells in addition to the traditional physical barrier.

In the aspect of cells and related exosomes, a previous study has displayed that degenerative AF cells could promote vascularization. Moon et al. (2014) have reported that AF cells from degenerative IVD stimulate endothelial cells and produce factors known to induce ECM degradation, angiogenesis, and innervation (Moon et al., 2014); degenerative NP cells have been proven to promote blood vessel growth by secreting pro-inflammatory factors (He et al., 2020). In the early stage of disc development, notochordal cells existing in NP cells could inhibit angiogenesis of IVD (Cornejo et al., 2015); additionally, a previous study has also provided evidence that degenerative AF exosomes could induce IVD vascularization and inflammation directly through upregulation of IL-6, TNF-α, and VEGF, and indirectly through enhancing the invasion of blood vessels *via* accumulation of MMPs (Sun et al., 2021). Furthermore, as for exosomes derived from MSC cells, a report indicated that miR-125a represses angiogenic inhibitor DLL4 in endothelial cells and thereby promotes angiogenesis (Liang et al., 2016). In terms of NP, although NP exosomes have been reported to exist and have numerous biological effects, they are mainly focused on stimulating MSC differentiation (Lan et al., 2019; Yuan et al., 2020); detailed research about NP exosomes and blood vessels remains limited. As a result, there is an urgent need to clarify the effects of NP exosomes on vascular endothelial cells.

PMSCs possess several characteristics such as proliferation, migration, cloning, and immune regulation, and have broad applications in clinical practice (Mathew et al., 2020). The numbers of cytokines and chemokines released from PMSC are a key point to modulate angiogenesis, which facilitates the possibility of considering PMSC exosomes as a target therapy to prompt angiogenesis. Liang et al. (2017) have reported that the co-culture system between PMSCs and EPCs enhances the angiogenic potential of EPCs through PDGF and the Notch signaling pathway, and conditioned media that may contain exosomes have significant pro-angiogenesis effects on EPCs and HUVECs (Komaki et al., 2017). PMSCs generate various kinds of angiogenic factors like VEGF, bFGF, IL-6, IL-8, and HGF (Komaki et al., 2017; Liang et al., 2017), and possible mechanisms of angiogenesis are also reported to include activation of PAKT and p38MAPK/pSTAT3 that induce VEGF secretion and recruitment of smooth muscle cells and pericytes (Chen et al., 2015; Makhoul et al., 2016). Several *in vivo* studies have gained promising results, namely, that PMSCs could enhance vessel density, blood flow, and perfusion in a dose- and site-dependent manner, especially in ischemia mice models (Francki et al., 2016; Xie et al., 2016; Zahavi-Goldstein et al., 2017). Interestingly, a comparative study (König et al., 2015) revealed that the angiogenic ability of MSCs derived from blood vessels is stronger than that in avascular sources, indicating the importance of determining the components of the conditioned medium from different cell sources. The above comments have fully discussed the mechanisms in terms of angiogenic potential, while in the aspect of anti-angiogenic potential of PMSCs, Alshareeda et al. (2018) have found that the co-culture of PMSCs along with breast cancer cells could significantly inhibit the migration, invasion, and tube formation ability of HUVECs. Zhang et al. (2014b) have shown that PMSCs could inhibit peritoneal tumorigenesis *via* downregulating blood vessel counts, and injection of PMSC into retinopathy mouse models has been demonstrated to prevent neovascularization through upregulation of TGF-β1 (Kim et al., 2016); these results could partly be explained by the upregulation of miRNAs like miR-136 under pathology situations that modulate the inhibition of capillary formation (Ji et al., 2017). Related studies about PMSC exosomes remain at an early stage; PMSC exosomes are known to modulate osteogenic and adipogenic differentiation by upregulating OCT4 and NANOG in dermal fibroblasts (Tooi et al., 2016), and they could enhance the migration and tube formation of endothelial cells (Komaki et al., 2017), while Yuan et al. (2020) have found the applications of PMSC exosomes in the treatment of IDD and that exosomes could upregulate ZNF121 and thus ameliorate IDD by delivering miR-4450 inhibitor (AntagomiR-4450). Basically, the applications of angiogenesis and anti-angiogenesis by PMSC exosomes are a controversial issue, modulated in a very contextual manner. More specific miRNAs from PMSC exosomes targeting IDD or endothelial cell functions as well as their potential roles are to be further identified.

## Prospects of Exosome Therapy for IDD

Accumulating lines of evidence have already reported the essential applications of different sources of exosomes in the treatment of IDD, which have been fully described in this review. Many studies have already demonstrated the role of exosomal miRNAs in inhibiting apoptosis of NP cells and suppressing MMP expression; however, the correlations among different sources of exosomes and whether the high expressed miRNAs in one kind of exosome are also detected in other exosomes remain unknown. These issues all need to be further addressed.

In addition to the inflammatory environment, the central portion of degenerative disc also has low cell density, low glucose, low pH value, low oxygen, high osmotic pressure, and high mechanical variations (Lu et al., 2017). Although MSC and MSC-derived exosomes have shown an effective influence on degenerative IVD, and cell-injection strategy has suggested promising results, there remain obstacles regarding MSC in clinical practice, especially how transplanted cells are able to survive and adapt in avascular IVD conditions and how to inhibit angiogenesis rather than promote blood vessel ingrowth of IVD when using exosomes resembling PMSC-derived (Krock et al., 2015; Sakai and Andersson, 2015); these issues are worth pondering and need to be resolved by scientific researchers.

Lastly, the most appropriate dose of exosome injection, along with the most optimal route of administration, remains unclear and requires further research (Loibl et al., 2019; Forsberg et al., 2020). The current studies about the administration route mainly focus on two points: direct intradiscal injection and systemic injection. Due to the avascular characteristics of IVD, it is hypothesized that direct injection of exosomes into the disc would be the most effective approach (Noriega et al., 2017), while an *in vivo* study of MSC exosomes administrated through tail vein by Zhang et al. (2020) also displayed promising results in the treatment of IDD. In the aspect of systemic injection, current studies only use single dose *in vivo*, and if systemic injections are applied, multiple doses may be conducted to maintain the therapeutic effects, and in this situation, it is essential to determine safety and efficiency, as well as the dose and frequency of injections. In terms of direct intradiscal injection, the following questions need to be addressed: Would the puncture needle for injection cause extra damage to the vertebral body or IVD? What is the definition of additional hurt to disc? Would the injection approach be applied in the clinic? To the best of our knowledge, there is only one clinical trial relating to the treatment of IDD by exosomes in India, which would be conducted when all participants are recruited. This clinical trial aims to observe the efficacy of PRP exosomes by intradiscal injection [Intra-discal Injection of Platelet-rich Plasma (PRP) Enriched With Exosomes in Chronic Low Back Pain, https://clinicaltrials.gov/ct2/show/NCT04849429]. Once these considerations are fully addressed, more clinical trials about IDD therapy using an exosome approach are needed.
